# Quantitative leukocyte *BDNF* promoter methylation analysis in bipolar disorder

**DOI:** 10.1186/2194-7511-1-28

**Published:** 2013-12-30

**Authors:** John S Strauss, Tarang Khare, Vincenzo De Luca, Richie Jeremian, James L Kennedy, John B Vincent, Arturas Petronis

**Affiliations:** Centre for Addiction and Mental Health, University of Toronto, Toronto, ON M6J1H4 Canada

**Keywords:** Neurotrophin, Methylation, Bipolar disorder, Epigenetic, Mass spectrometry

## Abstract

**Background:**

Bipolar disorder (BD) is a complex psychiatric phenotype with a high heritability and a multifactorial etiology. Multisite collaborative efforts using genome-wide association studies (GWAS) have identified only a portion of DNA sequence-based risk factors in BD. In addition to predisposing DNA sequence variants, epigenetic misregulation may play an etiological role in BD and account for monozygotic twin discordance, parental origin effects, and fluctuating course of BD. In this study, we investigated DNA methylation of the brain-derived neurotrophic factor (*BDNF*) gene in BD.

**Methods:**

Fifty participants with BD were compared to the same number of age- and sex-matched controls for DNA methylation differences at *BDNF* promoters 3 and 5. DNA methylation reads were obtained using a mass spectrophotometer for 64 cytosine-guanine (CpG) sites in 36 CpG ‘units’ across three amplicons of *BDNF* promoters 3 and 5.

**Results and Discussion:**

Methylation fractions differed between BD participants and controls for 11 of 36 CpG units. Five CpG units, mostly in promoter 5, remained significant after false discovery rate correction (FDR) (*p* values ≤ 0.004) with medium to large effect sizes (Cohen's *d* ≥ 0.61). Several of the significant CpGs overlapped with or were immediately adjacent to transcription factor binding sites (TFBSs) - including two of the FDR-significant CpG units in promoter 5. For the CpGs in promoter 3, there was a positive and significant correlation between age at sample collection and DNA methylation fraction (rho = 0.56, *p* = 2.8 ×10^−5^) in BD cases, but not in controls. Statistically significant differences in mean methylation fraction at 5/36 CpG units (after FDR), some at or immediately adjacent to TFBSs, suggest possible relevance for the current findings to BD etiopathogenesis. The positive correlation between age and methylation seen in promoter 3 is consistent with age-related decline in *BDNF* expression previously reported. Future studies should provide more exhaustive epigenetic study of the *BDNF* locus to better characterize the relationship between BDNF methylation differences and BD.

**Electronic supplementary material:**

The online version of this article (doi:10.1186/2194-7511-1-28) contains supplementary material, which is available to authorized users.

## Background

Bipolar disorder (BD) is a major psychiatric illness with a complex multifactorial etiology. Twin and adoption studies underscore the importance of inherited factors, and association studies, including genome-wide association studies by large consortia, have reported some replicable loci, notably *CACNA1C*, *ODZ4*, and *NCAN* (Cichon et al. 2011; Psychiatric GWAS Consortium Bipolar Disorder Working Group 2011). Despite the large sample sizes, family history in a close relative is still the best genetic prediction method for BD, and other molecular methods may be helpful to address the genetics of non-Mendelian disorders (Craddock and Sklar 2013; Schulze 2010).

In addition to genetic studies, discordance for BD in monozygotic (MZ) twins (Bertelsen et al. 1977; Sklar et al. 2002; McGuffin et al. 2003; Kieseppa et al. 2004) invites speculation about environmental risk factors. Evidence is suggestive of a role for environment in the genesis and course of BD (Serretti and Mandelli 2008). Prospective studies implicate psychosocial and other environmental variables in the timing of BD mood episodes (Proudfoot et al. 2011). Other data indicates that at least one third of adults with a BD diagnosis report childhood trauma, which is associated with more difficult course of illness (Leverich and Post 2006). Yet the specific role of environment remains unclear. Stressful life events are associated with BD, but robust evidence of a cause-effect relationship is lacking (Miklowitz and Chang 2008). And overall, environment plays a lesser role in the risk for developing the illness (McGuffin et al. 2003). Thus, despite the significant effort, much remains to be understood about the genetic and environmental basis of BD.

In recent years, epigenetic factors have become an avenue of investigation with some promise (Labrie et al. 2012), with the complex epigenetic regulation of *Bdnf* showing relevance to psychiatric disorders and environment (Boulle et al. 2012). Numerous clinical and epidemiological features of BD can be explained by epigenetic misregulation. Epigenetics refers to regulation of various genomic functions that are controlled by heritable, partially stable modifications in DNA methylation and/or chromatin structure (Henikoff and Matzke 1997). Epigenetic studies in twins have detected a large degree of MZ co-twin DNA methylation variation (Petronis et al. 2003; Kaminsky et al. 2009), which may contribute to the MZ twin discordance observed in BD. Putative epigenetic misregulation is also consistent with variable age of onset, fluctuating clinical course with exacerbations and remissions, peaks of susceptibility coinciding with hormonal changes, parent-of-origin effects, and sexual dimorphism (Arnold et al. 2003).

Epigenetics gives additional framework for understanding the function of genome sequence and genetic complexity. Ambiguous genetic results on complex disease phenotypes could be more salient if considered in an epigenomic context (Feinberg 2010). In aggregate, such findings have led to the notion that epigenetic factors may be relevant to complex non-Mendelian phenotypes like BD (Labrie et al. 2012; Petronis 2003) and may account for a fraction of the ‘missing heritability’ of complex traits (Maher 2008).

Several lines of evidence link brain-derived neurotrophic factor (BDNF) to BD. Serum BDNF levels are reduced in depression (Molendijk et al. 2013), euthymic BD (Monteleone et al. 2008), acute mania (Machado-Vieira et al. 2007; Tramontina et al. 2009), and bipolar depression (Fernandes et al. 2009) and are lower with longer duration of illness (Kauer-Sant'Anna et al. 2009). A meta-regression has confirmed that serum/plasma BDNF levels are consistently reduced during manic and depressive episodes and are restored to normal levels in subjects treated for acute mania (*n* = 1,113 subjects) across 13 studies (Fernandes et al. 2011). From genetics reports, polymorphisms within the BDNF gene have been studied in BD (Neves-Pereira et al. 2002; Sklar et al. 2002). There is a frequently cited association between BD and the SNP rs6265, also known as the Val66Met variant. Association of SNP rs6265 has been repeatedly shown with BD and was statistically significant in a meta-analysis of 14 studies comprising of 4,248 cases, 7,080 controls, and 858 nuclear families (Fan and Sklar 2008). More recently, rs6265 Met allele carriers were observed to have different serum BDNF profiles during a period of treatment than Val allele homozygotes (Grande et al. 2013).

A limited number of studies have been conducted to investigate DNA modifications at *BDNF* in BD, and most were done on postmortem brain tissue. Among them, a comprehensive study showed cytosine-guanine dinucleotide (CpG) DNA methylation variation in BD patients in proximity of the *BDNF* SNP rs6265; exonic CpG methylation was associated with the valine variant of rs6265 in a sample of 105 postmortem brains (Mill et al. 2008). In another study, Rao et al. (2012) demonstrated increased methylation of *BDNF* promoter 1 in frontal cortex tissue in ten BD brains compared to ten age-matched controls (*p* < 0.05). Hypermethylation at *BDNF* promoter 1 in peripheral blood monocyte DNA has also been reported in 16 participants with Bipolar II Disorder (BD-II) (*p* < 0.01) but not for 16 participants with Bipolar I Disorder (BD-I) when compared to controls; the investigators also observed that lithium and valproate reduce promoter 1 DNA methylation (D'Addario et al. 2012).

The evidence reviewed above suggests a putative role for the *BDNF* locus in BD. Our aim was to characterize epigenetic regulation of *BDNF* in peripheral blood, and our hypothesis was that BD patients exhibit *BDNF* promoter methylation differences compared to controls.

## Methods

### Subjects

Participants consisted of 50 randomly selected BD cases and 50 unrelated healthy controls matched for age and sex from a larger sample (*n* = 452 BD cases) as previously described (Scott et al. 2009; Psychiatric GWAS Consortium Bipolar Disorder Working Group 2011). Inclusion criteria were as follows: (a) diagnosed with DSM-IV or ICD-10 BD-I or II, (b) age 18 years old or above, and (c) Caucasian, of Northern and Western European origin. Exclusion criteria includes the following: (a) diagnosis of intravenous drug dependence or reported use of intravenous drugs, (b) evidence of mental retardation, (c) related to an individual already in the study, (d) manias *that only* ever occurred in relation to or as a result of alcohol or substance abuse or dependence, medications, and/or medical illness, and (e) had mood-incongruent psychotic symptoms. BD diagnoses were established according to DSM-IV or ICD-10 criteria, using the computerized algorithm (CATEGO) for the SCAN 2.1 interview (WHO) (Celik 2003). Our investigation was completed in compliance with the Declaration of Helsinki and was approved by the Research Ethics Board of the Centre for Addiction and Mental Health.

#### Clinical and demographic characteristics

Fifty randomly selected cases were included. All cases selected had BD-I; none of the 50 randomly selected cases had BD-II. All participants were of northern European ancestry. They were equal with equal numbers of males and females. Cases and controls were matched for sex and age. Mean ages at phlebotomy (SD) for cases and controls were 45.3 (12.4) and 45.4 (12.4) years, respectively.

### Genomic DNA extraction

Venous blood was collected in Toronto at the Centre for Addiction and Mental Health and preserved in ethylenediaminetetraacetic acid (1.8 mg EDTA/mL of blood). DNA was extracted using a high salt method (Lahiri and Nurnberger 1991). Quantity and quality of genomic DNA was estimated on nanodrop spectrophotometer (Nanodrop Products, Wilmington, DE, USA), and genomic DNA with 260/280 ratio >1.8 and 260/230 >1.9 was taken for downstream analysis.

### Estimation of *BDNF* promoter DNA methylation

**Figure 1:**
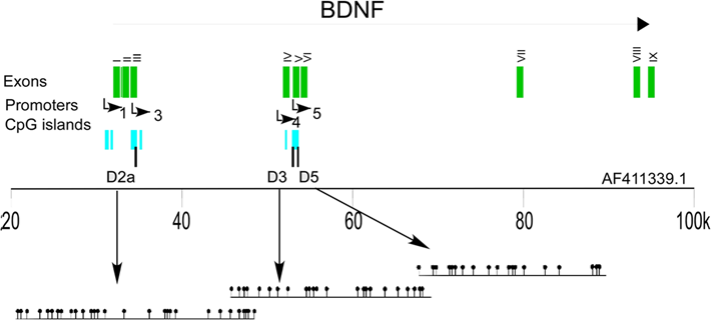
**Graphical overview at BDNF gene.** The BDNF gene structure and promoter locations are adopted from the study of Pruunsild et al. (2007). Arrow on the top shows the direction of BDNF transcript; exons (green), CpG islands (cyan), and investigated regions for DNA methylation (black) are mapped on NCBI accession number AF411339.1. CpG island were detected by CpGPlot using default parameters (http://www.ebi.ac.uk/Tools/seqstats/emboss_cpgplot/). Number of CpG dinucleotides in each amplicons is shown as lollipops on the lower panel.

*BDNF* sequence (AF411339.1) was used to map different known CpG promoters within the gene (Figure 1); two methods were used. First, the CSHL human promoter mapping using CpG islands (CpGpromoter) (Ioshikhes and Zhang 2000) enabled mapping of human promoters using Gardiner-Garden and Frommer's definition of CpG islands (Gardiner-Garden and Frommer 1987) (http://rulai.cshl.org/tools/CpG_promoter). CpGpromoter indicated CpG islands related to promoters at three regions of AF411339.1. Second is the BIMAS Promoter Scan (Center for Information Technology, NIH, http://www-bimas.cit.nih.gov/molbio/proscan/), which uses PROSCAN 1.7 to predict promoters based on scoring homologies with eukaryote Pol II sequences. BIMAS corroborated the three CpG island promoters noted with CpGpromoter, which correspond to *BDNF* promoters 1, 3, and 5 of the nine promoters described by Pruunsild et al. (2007) (see Figure 1).

Bisulfite conversion was performed on 500 ng of genomic DNA using the QiagenEpiTect Kit, Hilden, Germany, according to the manufacturer’s protocol. Primers were then designed on these promoters, and in total, three amplicons were generated covering 30 CpG sites at promoter 3 and 42 CpG sites at promoter 5.

Multiple attempts and different primer pairs in our hands failed to amplify any region at promoter 1. Bisulfite primers and polymerase chain reaction (PCR) amplification conditions are stated in Additional file 1: Table S1.

The amplified fragments were analyzed on a MassARRAY platform (Sequenom, San Diego, CA, USA) at the Clinical Genomics Centre in Toronto (http://www.clinicalgenomics.ca/). Locus-specific PCR amplification was performed with the T7-promoter tagged primers, where the latter was used to generate *in vitro* transcription on the amplified fragments. These transcripts were then subjected to enzymatic RNA base pair cleavage. The resulting fragments differ in size and mass depending on the sequence changes generated through bisulfite treatment. The fragment mass was determined by matrix-assisted laser desorption/ionization time-of-flight (MALDI-TOF) mass spectrometry; then, EpiTYPER software converted MALDI-TOF (Ehrich et al. 2005) values to quantitative percent of CpG dinucleotide methylation. The method reads small DNA fragments for mass-to-charge ratio, and these small reads may map to multiple CpG sites on the same amplicon; hence, often two or three CpG sites have one measured value. The resulting analytic units are called *CpG units* which consist of a single CpG site or a combination of two or three CpG sites. Methylation measures were performed in triplicate expressed as a methylation fraction (range 0 to 1) for each CpG.

Additional exploratory analyses of *BDNF* promoter methylation by *BDNF* rs6265 (i.e., Val66Met) genotype were conducted to investigate possible interaction effects. Genotyping with Illumina HumanHap550 BeadChip, Illumina Inc., San Diego, CA, USA, and quality control methods were as previously described (Scott et al. 2009). Specifically, individual CpG unit methylation was tested for association with BD in two genotype groups: A (rare) allele carriers consisting of AG and AA genotypes and G (common) allele homozygotes.

### Statistical analysis

Univariate statistics were performed for demographic and clinical characteristics of BD patients and control individuals. The means of the triplicate methylation fractions (MMFs) were examined, only the readouts with standard deviations < 0.15; therefore, 8 of 72 CpG sites were excluded for a total of 64 CpGs comprising 36 CpG units. Since 15 of 36 CpG units had significant differences in homogeneity of variances, the nonparametric Mann–Whitney test was used for differences in mean methylation fractions (in both Table 1 and Additional file 2: Table S2). We tested age and sex for association with DNA methylation and corrected for multiple testing with false discovery rate (FDR) (Benajmini and Hochberg 1995) for 36 CpG units, yielding a significance threshold of alpha = 0.004.

**Table 1:** **Mean methylation fractions for BDNF CpG units in bipolar disorder versus controls**

Amplicon	CpG unit	BP MMF (SD)	Controls MMF (SD)	***Z***	Asymp. sig.	Cohen's ***d***
D2a	1, 2	0.354 (0.077)	0.335 (0.067)	−1.712	0.087	
	11, 26	0.156 (0.025)	0.159 (0.038)	−0.061	0.951	
	12, 13, 14	0.301 (0.030)	0.289 (0.034)	−1.864	0.062	
	15	0.103 (0.022)	0.100 (0.026)	−0.682	0.495	
	16	0.086 (0.012)	0.082 (0.015)	1.797	0.072	
	17	0.122 (0.032)	0.116 (0.015)	−2.33	0.02*	
	18, 19, 20	0.242 (0.032)	0.232 (0.035)	−1.789	0.072	
	21	0.128 (0.014)	0.126 (0.023)	−0.449	0.654	
	22, 23	0.275 (0.018)	0.260 (0.023)	−2.094	0.036*	
	24	0.089 (0.011)	0.086 (0.028)	−1.217	0.224	
	27, 28, 29, 30	0.246 (0.031)	0.238 (0.014)	−1.284	0.199	
	3	0.355 (0.034)	0.333 (0.032)	−3.315	*0.001* **	0.680
	4	0.054 (0.025)	0.048 (0.031)	−1.054	0.292	
	5, 6	0.279 (0.047)	0.274 (0.022)	−0.733	0.464	
	9, 10	0.254 (0.034)	0.255 (0.038)	−0.256	0.798	
D3	11, 12, 13	0.227 (0.014)	0.217 (0.019)	−2.889	*0.004* **	0.610
	14	0.085 (0.011)	0.079 (0.017)	−2.37	0.018*	
	15, 16, 17	0.181 (0.016)	0.163 (0.027)	−2.993	*0.003* **	0.840
	18	0.068 (0.012)	0.061 (0.011)	−3.179	*0.001* **	0.610
	19	0.055 (0.011)	0.056 (0.012)	−0.157	0.875	
	2, 3, 4	0.284 (0.019)	0.282 (0.020)	−0.484	0.628	
	20	0.480 (0.068)	0.504 (0.101)	−0.728	0.466	
	21	0.021 (0.008)	0.021 (0.015)	−0.728	0.466	
	22, 23, 8	0.136 (0.008)	0.126 (0.014)	−3.8	*<0.001* **	0.910
	5, 6	0.316 (0.029)	0.302 (0.054)	−0.601	0.548	
	7	0.068 (0.011)	0.066 ()0.016	−0.799	0.424	
	9, 10	0.069 (0.009)	0.066 (0.012)	−2.017	0.044*	
D5	11, 12, 13	0.233 (0.030)	0.220	−1.962	0.05*	
	14	0.065 (0.014)	0.063	−0.853	0.394	
	15	0.253 (0.085)	0.253	−0.155	0.877	
	16	0.093 (0.022)	0.083	−2.119	0.034*	
	17, 18, 19	0.172 (0.017)	0.160	−2.447	0.014*	
	4, 5	0.168 (0.097)	0.200	−1.09	0.276	
	6	0.056 (0.017)	0.057	−0.391	0.696	
	7, 8	0.205 (0.021)	0.197	1.671	0.095	
	9	0.061 (0.048)	0.074	−1.35	0.177	

*Significant at alpha = 0.05; **significant after FDR, alpha = 0.004; BP, bipolar disorder. MMF, mean methylation; *d,* effect size; Z, Mann Whitney U test statistic.

## Results

### Regional CpG promoter methylation

We examined DNA methylation for each CpG promoter region using the average of all CpG units mean methylation fractions (MMFs) in a *BDNF* region that we studied, in this case, the amplicons that were assayed. A significant difference in *BDNF* methylation between cases and controls for region/amplicon D3 (*Z* = −2.185, *p* = 0.029) was noted. The other two regions exhibited no significant case–control differences in DNA methylation, D2a (*Z* = −1.915, *p* = 0.055) or D5 (*Z* = −0.0417, 0.677). This was also evident at the individual CpG level of DNA methylation analysis (below), where the majority of significant CpG units were in region D3.

### Methylation of individual CpGs

#### D2a amplicon

Individual CpG units with nominal (*p* < 0.05) case–control differences in MMF were CpG 17 (*Z* = −2.33, *p* = 0.02), CpG 22,23 (*Z* = −2.094, *p* = 0.036), and CpG 3 (*Z* = −3.315, *p* = 0.001. Cases were more methylated than controls; of these, only CpG 3 passed FDR correction).

#### D3 amplicon

Several CpG units demonstrated significance within this region. Four CpG units survived FDR, including CpG 11,12,13 (*Z* = −2.889, *p* = 0.004), CpG 15,16,17 (*Z* = −2.993, *p* = 0.003), CpG 18 (*Z* = −3.179, *p* = 0.001), and CpG 22,23,8 (*Z* = −3.8, *p* = 0.001). Two CpG units had nominally significant case–control differences in methylation, CpG 14 (*Z* = −2.37, *p* = 0.018) and 9,10 (Z = −2.017, *p* = 0.044); at each of the significant CpG units, cases had higher MMFs than controls.

#### D5 amplicon

Differences in methylation were nominally significant at CpG units 11,12,13 (*Z* = −1.962, *p* = 0.05), 16 (*Z* = −2.119, *p* = 0.034), and 17,18,19 (*Z* = −2.447, *p* = 0.014); none were significant after the FDR correction.

For CpG units which remained significant after FDR, differences in MMF ranged from 0.007 to 0.022, with Cohen's *d* ranging from 0.61 to 0.91. See Table 1.

### TFBS mapping

Next, we looked for the transcription factor binding sites (TFBSs) that are in proximity to significant individual CpG results. For this, we mapped the DNA sequences of the three *BDNF* amplicons for their TFBSs, using the transcription factor binding profile database JASPAR (http://jaspar.genereg.net/), and investigated the distance between CpGs having at least nominally significant group differences in DNA methylation and the nearest TFBS. At the D2a amplicon, CpG unit 17 showed GATA binding protein 2 and 3 (GATA2 and GATA3) TFBSs. For amplicon D3, CpG unit 15,16,17 showed a complete overlap with an AP-2 alpha (activating enhancer binding protein 2 alpha) (TFAP2A) (Comb and Goodman 1990) binding site, and another TFAP2A site was within 3 bp downstream of CpG unit 22,23,8. Similarly, within amplicon D5, three TFBSs were at or within 15bp of CpGs: a TFAP2A site spans CpG unit 11,12,13; a myeloid zinc finger 1 (MZF1) TFBS was at CpG unit 17,18,19, and a nuclear factor-kappa B (NFKB1) (Rau et al. 2012) TFBS was 15 bp downstream of CpG 17,18,19. The TFBSs above - GATA2, GATA3, TFAP2A, MZF1, and NFKB1 - are all expressed in brain and white blood cells.

### Age, sex, and BDNF methylation

**Figure 2:**
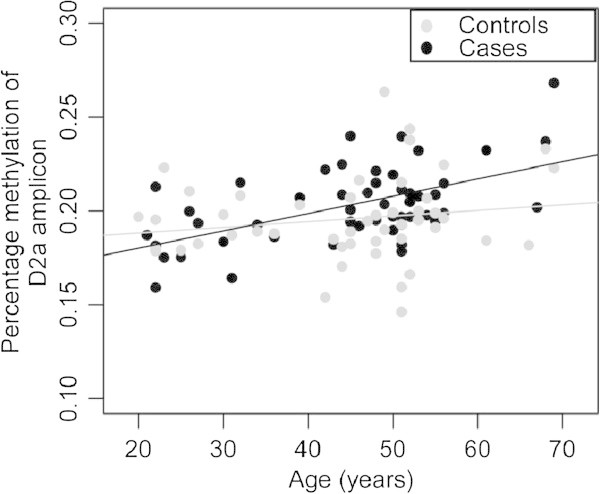
**Correlation between age and CpG unit methylation.** Methylation at amplicon D2a is correlated with age in bipolar cases but not in controls. Black, BP cases (rho = 0.56, *p* = 2.80 × 10^−5^). Gray, controls (rho = 0.17, *p* = 0.25).

The D2a amplicon showed a positive correlation between age at sample collection and MMF (rho = 0.56, *p* = 2.80 × 10^−5^) in BD cases, but no correlation was observed in controls (rho = 0.17, *p* = 0.25) (Figure 2). In contrast, no correlation of DNA methylation with age was observed for D3 or D5 amplicons. No sex effects on DNA methylation were observed in any of the three amplicons (see Figure 2).

In summary, we found five CpG units in *BDNF* promoters with MMFs differing between BD and controls, predominantly in the D3 amplicon. We also observed positive correlation between age and MMF in D2a amplicon of promoter 3, significant in cases but not controls.

When testing for genotype group differences at individual CpG unit methylation sites, six CpG units were nominally significant (two in each of the three amplicons) at alpha = 0.05. With a slightly more stringent threshold of alpha = 0.01, six other CpG units remained significant, five of which were in amplicon D3. The preponderance of most significant *post hoc* methylation findings were with the rs6265 G or Val homozygotes at CpG sites that were significant after FDR in the initial BD control analysis (D2, CpG unit 3; D3, CpG units 11,12,13; 15,16,17; 22,23,8). See Additional file 2: Table S2.

## Discussion

We examined three amplicons over two *BDNF* CpG promoters in 50 BD I participants and 50 age- and sex-matched controls. Of 36 CpG units, 6 CpG units differed nominally in MMF between cases and controls; 5 CpG units’ MMFs, mostly in amplicon D3 (promoter 5), remained significant following FDR. Effect sizes were medium to large (0.61 to 0.91) at CpG units significant after FDR, while absolute differences in methylation fraction were small (0.002 to 0.022). We noted that several of these significant CpG units were at or in the immediate vicinity of TFBSs. We also observed a statistically significant correlation between the participant's age and DNA methylation for D2a (promoter 3) in the cases; however no correlation was found for the controls. The majority of findings published to date have been on other promoters, unlike ours, which focused on promoters 3 and 5. The age promoter 3 methylation correlation was less strong when cases and controls were pooled (rho = 0.353; *p* = 0.00036) but still significant.

Earlier investigations have examined *BDNF* methylation in affected brain tissues. In *postmortem* tissue from Wernicke's area, four CpG dinucleotides in the *BDNF* promoter 4 area had elevated methylation in suicide cases compared to controls (Keller et al. 2010). Another *postmortem* brain study reported *BDNF* promoter hypermethylation in frontal cortex tissue in BD (Rao et al. 2012), in accord with the same group's previous finding of reduced *BDNF* mRNA in Brodmann area 9 of BD brains (Kim et al. 2010). The investigators examined several loci in the same region in BD and Alzheimer's disease, including 81 CpG sites in *BDNF* promoter 1. Our findings differ not only in the tissue of interest but also in the promoters assayed.

A limited number of studies have focused the DNA methylation analysis at *BDNF* promoters using blood-derived DNA in psychiatric or addiction patients. For example, maternal prenatal smoking has been shown to increase methylation of *BDNF* exon 6 in adolescent offspring (Toledo-Rodriguez et al. 2010). In major psychosis patients, *BDNF* promoter 1 was hypermethylated in peripheral blood monocytes for BD-II but not for BD-I; again, we did not assay promoter 1 and have no methylation data on BP-II for the current report. Influence from medication has also been reported: antidepressants were associated with increased methylation, while mood stabilizers were associated with reduced methylation (D'Addario et al. 2012). The CpG units that were significant in our investigation had cases with higher MMFs than controls. *BDNF* promoter 1 DNA methylation signals were also reported for unipolar major depressive disorder patients; a small number of CpG units demonstrated several fold increase or decrease in methylation in cases versus controls by mass spectrometry and subsequent hierarchical cluster analysis, generating *p* values smaller than 10^−11^ (Fuchikami et al. 2011). Other studies have recently demonstrated higher degrees of methylation in the *BDNF* region to be associated with other mood-related phenotypes such as poststroke depression (Kim et al. 2013) and antidepressant response (Tadić et al. 2013).

Association of age with DNA methylation, including promoter methylation, is well documented (Jaenisch and Bird 2003; Kwabi-Addo et al. 2007; Vasilatos et al. 2009; Liu et al. 2010). Human prefrontal cortex genomic DNA has age-related dynamic methylation at several gene promoters (Numata et al. 2012). DNA methylation in blood cells was also reported to correlate with age and local sequence features (Langevin et al. 2011). Some studies conflict or suggest that methylation-age associations are tissue- and locus-dependent (Eckhardt et al. 2006; Heijmans et al. 2007). We noted a positive correlation between age and CpG methylation in *BDNF* promoter 3, only for the BD cases. Serum BDNF concentrations have been negatively correlated with age (Kauer-Sant’Anna et al. 2009). Taken together, such findings suggest that a reduction in BDNF expression with age might be associated with increased methylation observed at promoter 3.

Some limitations in the present study require acknowledgement. First, we opted for an agnostic *in silico* approach for CpG promoter detection with CpGpromoter and BIMAS and investigated a few promoters. There are nine known *BDNF* promoters. We created primers for three (promoters 1, 3, and 5 for reasons explained above and immediately below), one of which (promoter 1) was not completed due to technical issues. Promoters other than 1, 3, and 5 did not score as highly on the in silico scans.

Our laboratory method for DNA methylation investigation exploits mass spectrometry, which is a precise technique that detects very small DNA methylation differences (to 14 kDa). This highly sensitive method was used to assay candidate CpG units from peripheral blood DNA and hence requires validation for DNA methylation differences in the affected tissue, i.e., *postmortem* brain. While some evidence points to differences between blood, cortical, and cerebellar methylation for several neurodevelopmental genes, including *BDNF* (Davies et al. 2012), generally, *BDNF* methylation shows consistency across brain and blood (Ikegame et al. 2013). Still, a growing number of analyses based on leukocyte-derived DNA methylation in psychiatric disorders exist, including a study of the DRD2 5′-regulatory region in sib pairs discordant for schizophrenia (Zhang et al. 2007), a functional study of the *SLC12A6* promoter (Moser et al. 2009), and a methylation-sensitive representational difference analysis of lymphoblastoid cells derived from monozygotic twins noted above (Kuratomi et al. 2008), the report on *BDNF* methylation in unipolar depression noted above (Fuchikami et al. 2011), and the investigation of BD (D'Addario et al. 2012). Another limitation is that the approach we used, namely bisulfite conversion-based analysis, is not able to differentiate 5-methyl cytosine (5-mC) from 5-hydroxymethyl cytosine (5-hmC) and unmodified cytosine from 5-formyl cytosine (5-fC) plus 5-carboxylcytosine (5-caC). A final limitation is that medication effects may be causing false positive results; we lacked the required current mood stabilizer data to characterize such potential influences on methylation.

Here, we also report exploratory *post hoc* results showing that a majority (4/5) of the FDR-significant methylation signals from the initial analysis were statistically significant in rs6265 Val homozygotes (GG) and not in Met carriers (AG+AA). Although the findings are not without some ambiguity, they are suggestive of SNP-methylation interactions.

Meta-analysis of the original 14 published studies of rs6265 in BD, including family-based and case–control designs, showed moderate nominal association for rs6265 - specifically the G(Val) allele was associated with BD. Though much evidence points to the rare Met allele having deleterious cognitive effects, there has been curiosity why the common Val allele is the one associated with BD (Fan and Sklar 2008). Our preliminary results in this small sample, if confirmed by replication, suggest rs6265 SNP-promoter methylation interactions may be measurable in BD.

## Conclusions

The sample size is small and therefore underpowered to draw firm conclusions. Our results add to a growing literature on *BDNF* methylation in BD by the use of precise mass spectrometry assays of CpGs in previously uninvestigated *BDNF* promoters in one of the largest BD epigenetics samples of peripheral tissue to date; they underscore the complexity of *BDNF* regulation across nine functional promoters and multiple transcripts and altogether suggest a need for more comprehensive epigenetic investigation of the locus.

## Electronic supplementary material

Additional file 1: Table S1: PCR, SAP and cleavage conditions, with primers. (DOC 34 KB)

Additional file 2: Table S2: Methylation results by *BDNF* rs6265 genotype. (DOC 40 KB)

## Abbreviations

BD: Bipolar disorder
BD-I: Bipolar I Disorder
BD-II: Bipolar II Disorder
BDNF: Brain-derived neurotrophic factor
CpG: Cytosine-guanine dinucleotide
GATA2: GATA binding protein 2
GATA3: GATA binding protein 3
MMF: Mean methylation fraction
MZF1: Myeloid zinc finger 1
NFKB1: Nuclear factor of kappa light polypeptide gene enhancer in B cells 1
PCR: Polymerase chain reaction
SCAN: Schedules for clinical assessment in neuropsychiatry
TFAP2A: Transcription factor AP-2 alpha
TFBS: Transcription factor binding site.

## References

[CR1] Arnold AP, Rissman EF, De Vries GJ (2003). Two perspectives on the origin of sex differences in the brain. Ann N Y Acad Sci.

[CR2] Benajmini Y, Hochberg Y (1995). Controlling the false discovery rate: a practical and powerful approach to multiple testing. J R Stat Soc Series B Stat Methodol.

[CR3] Bertelsen A, Harvald B, Hauge M (1977). A Danish twin study of manic-depressive disorders. Br J Psychiatry.

[CR4] Boulle F, van den Hove DL, Jakob SB, Rutten BP, Hamon M, van Os J, Lesch KP, Lanfumey L, Steinbusch HW, Kenis G (2012). Epigenetic regulation of the BDNF gene: implications for psychiatric disorders. Mol Psychiatry.

[CR5] Celik C (2003). Computer Assisted Personal Interviewing Application for the Schedules for Clinical Assessment in Neuropsychiatry Version 2.1 and Diagnostic Algorithms for WHO ICD 10 chapter V DCR and for Diagnostic and Statistical Manual of Mental Disorders IV. Release 1 Ed 1.0.3.5 Win9xNT.

[CR6] Cichon S, Muhleisen TW, Degenhardt FA, Mattheisen M, Miró X, Strohmaier J, Steffens M, Meesters C, Herms S, Weingarten M, Priebe L, Haenisch B, Alexander M, Vollmer J, Breuer R, Schmäl C, Tessmann P, Moebus S, Wichmann HE, Schreiber S, Müller-Myhsok B, Lucae S, Jamain S, Leboyer M, Bellivier F, Etain B, Henry C, Kahn JP, Heath S, Bipolar Disorder Genome Study (BiGS) Consortium (2011). Genome-wide association study identifies genetic variation in neurocan as a susceptibility factor for bipolar disorder. Am J Hum Genet.

[CR7] Comb M, Goodman HM (1990). CpG methylation inhibits proenkephalin gene expression and binding of the transcription factor AP-2. Nucleic Acids Res.

[CR8] Craddock N, Sklar P (2013). Genetics of bipolar disorder. Lancet.

[CR9] D'Addario C, Dell'Osso B, Palazzo MC, Benatti B, Lietti L, Cattaneo E, Galimberti D, Fenoglio C, Cortini F, Scarpini E, Arosio B, Di Francesco A, Di Benedetto M, Romualdi P, Candeletti S, Mari D, Bergamaschini L, Bresolin N, Maccarrone M, Altamura AC (2012). Selective DNA methylation of BDNF promoter in bipolar disorder: differences among patients with BDI and BDII. Neuropsychopharmacol.

[CR10] Davies MN, Volta M, Pidsley R, Lunnon K, Dixit A, Lovestone S, Coarfa C, Harris RA, Milosavljevic A, Troakes C, Al-Sarraj S, Dobson R, Schalkwyk LC, Mill J (2012). Functional annotation of the human brain methylome identifies tissue-specific epigenetic variation across brain and blood. Genome Biol.

[CR11] Eckhardt F, Lewin J, Cortese R, Rakyan VK, Attwood J, Burger M, Burton J, Cox TV, Davies R, Down TA, Haefliger C, Horton R, Howe K, Jackson DK, Kunde J, Koenig C, Liddle J, Niblett D, Otto T, Pettett R, Seemann S, Thompson C, West T, Rogers J, Olek A, Berlin K, Beck S (2006). DNA methylation profiling of human chromosomes 6, 20 and 22. Nat Genet.

[CR12] Ehrich M, Nelson MR, Stanssens P, Zabeau M, Liloglou T, Xinarianos G, Cantor CR, Field JK, van den Boom D (2005). Quantitative high-throughput analysis of DNA methylation patterns by base-specific cleavage and mass spectrometry. Proc Natl Acad Sci U S A.

[CR13] Fan J, Sklar P (2008). Genetics of bipolar disorder: focus on BDNF Val66Met polymorphism. Novartis Found Symp.

[CR14] Feinberg AP (2010). Epigenomics reveals a functional genome anatomy and a new approach to common disease. Nat Biotechnol.

[CR15] Fernandes BS, Gama CS, Kauer-Sant’Anna M, Lobato MI, Belmonte-de-Abreu P, Kapczinski F (2009). Serum brain-derived neurotrophic factor in bipolar and unipolar depression: a potential adjunctive tool for differential diagnosis. J Psychiatr Res.

[CR16] Fernandes BS, Gama CS, Ceresér KM, Yatham LN, Fries GR, Colpo G, de Lucena D, Kunz M, Gomes FA, Kapczinski F (2011). Brain-derived neurotrophic factor as a state-marker of mood episodes in bipolar disorders: a systematic review and meta-regression analysis. J Psychiatr Res.

[CR17] Fuchikami M, Morinobu S, Segawa M, Okamoto Y, Yamawaki S, Ozaki N, Inoue T, Kusumi I, Koyama T, Tsuchiyama K, Terao T (2011). DNA methylation profiles of the brain-derived neurotrophic factor (BDNF) gene as a potent diagnostic biomarker in major depression. Plos One.

[CR18] Gardiner-Garden M, Frommer M (1987). CpG islands in vertebrate genomes. J Mol Biol.

[CR19] Grande I, Magalhaes PVS, Chendo I, Stertz L, Fries GR, Cereser KM, Cunha AB, Gói P, Kunz M, Udina M, Martín-Santos R, Frey BN, Vieta E, Kapczinski F (2013). Val66Met polymorphism and serum brain-derived neurotrophic factor in bipolar disorder: an open-label trial. Acta Psychiatr Scand.

[CR20] Heijmans BT, Kremer D, Tobi EW, Boomsma DI, Slagboom PE (2007). Heritable rather than age-related environmental and stochastic factors dominate variation in DNA methylation of the human IGF2/H19locus. Human Mol Genet.

[CR21] Henikoff S, Matzke MA (1997). Exploring and explaining epigenetic effects. Trends Genet.

[CR22] Ikegame T, Bundo M, Murata Y, Kasai K, Kato T, Iwamoto K (2013). Methylation of the BDNF gene and its relevance to psychiatric disorders. J Hum Genet.

[CR23] Ioshikhes IP, Zhang MQ (2000). Large-scale human promoter mapping using CpG islands. Nat Genet.

[CR24] Jaenisch R, Bird A (2003). Epigenetic regulation of gene expression: how the genome integrates intrinsic and environmental signals. Nat Genet.

[CR25] Kaminsky ZA, Tang T, Wang S-C, Ptak C, Oh G, Wong AH, Feldcamp LA, Virtanen C, Halfvarson J, Tysk C, McRae AF, Visscher PM, Montgomery GW, Gottesman II, Martin NG, Petronis A (2009). DNA methylation profiles in monozygotic and dizygotic twins. Nat Genet.

[CR26] Kauer-Sant’Anna M, Kapczinski F, Andreazza AC, Bond DJ, Lam RW, Young LT, Yatham LN (2009). Brain-derived neurotrophic factor and inflammatory markers in patients with early- vs. late-stage bipolar disorder. Int J Neuropsychopharmacol.

[CR27] Keller S, Sarchiapone M, Zarrilli F, Videtic A, Ferraro A, Carli V, Sacchetti S, Lembo F, Angiolillo A, Jovanovic N, Pisanti F, Tomaiuolo R, Monticelli A, Balazic J, Roy A, Marusic A, Cocozza S, Fusco A, Bruni CB, Castaldo G, Chiariotti L (2010). Increased BDNF promoter methylation in the Wernicke area of suicide subjects. Arch Gen Psychiatry.

[CR28] Kieseppa T, Partonen T, Haukka J, Kaprio J, Lönnqvist J (2004). High concordance of bipolar I disorder in a nationwide sample of twins. Am J Psychiatry.

[CR29] Kim HW, Rapoport SI, Rao JS (2010). Altered expression of apoptotic factors and synaptic markers in postmortem brain from bipolar disorder patients. Neurobiol Dis.

[CR30] Kim JM, Stewart R, Kang HJ, Kim SY, Kim SW, Shin IS, Park MS, Kim HR, Shin MG, Cho KH, Yoon JS (2013). A longitudinal study of BDNF promoter methylation and genotype with poststroke depression. J Affect Disord.

[CR31] Kuratomi G, Iwamoto K, Bundo M, Kusumi I, Kato N, Iwata N, Ozaki N, Kato T (2008). Aberrant DNA methylation associated with bipolar disorder identified from discordant monozygotic twins. Mol Psychiatry.

[CR32] Kwabi-Addo B, Chung W, Shen L, Ittmann M, Wheeler T, Jelinek J, Issa JP (2007). Age-related DNA methylation changes in normal human prostate tissues. Clin Cancer Res.

[CR33] Labrie V, Pai S, Petronis A (2012). Epigenetics of major psychosis: progress, problems and perspectives. Trends Genet.

[CR34] Lahiri DK, Nurnberger JI (1991). A rapid non-enzymatic method for the preparation of HMW DNA from blood for RFLP studies. Nucleic Acids Res.

[CR35] Langevin SM, Houseman EA, Christensen BC, Wiencke JK, Nelson HH, Karagas MR, Marsit CJ, Kelsey KT (2011). The influence of aging, environmental exposures and local sequence features on the variation of DNA methylation in blood. Epigenetics.

[CR36] Leverich GS, Post RM (2006). Course of bipolar illness after history of childhood trauma. Lancet.

[CR37] Liu M, Peng Y, Wang X, Guo Q, Shen S, Li G (2010). NGX6 gene mediated by promoter methylation as a potential molecular marker in colorectal cancer. BMC Cancer.

[CR38] Machado-Vieira R, Dietrich MO, Leke R, Cereser VH, Zanatto V, Kapczinski F, Souza DO, Portela LV, Gentil V (2007). Decreased plasma brain derived neurotrophic factor levels in unmedicated bipolar patients during manic episode. Biol Psychiatry.

[CR39] Maher B (2008). The case of the missing heritability. Nature.

[CR40] McGuffin P, Rijsdijk F, Andrew M, Sham P, Katz R, Cardno A (2003). The heritability of bipolar affective disorder and the genetic relationship to unipolar depression. Arch Gen Psychiatry.

[CR41] Miklowitz DJ, Chang KD (2008). Prevention of bipolar disorder in at-risk children: theoretical assumptions and empirical foundations. Dev Psychopathol.

[CR42] Mill J, Tang T, Kaminsky Z, Khare T, Yazdanpanah S, Bouchard L, Jia P, Assadzadeh A, Flanagan J, Schumacher A, Wang SC, Petronis A (2008). Epigenomic profiling reveals DNA-methylation changes associated with major psychosis. Am J Hum Genet.

[CR43] Molendijk ML, Spinhoven P, Polak M, Bus BAA, Penninx BWJH, Elzinga BM (2013). BDNF concentrations as peripheral manifestations of depression: evidence from a systematic review and meta-analyses on 179 associations (N 1⁄4 9484). Mol Psychiatry.

[CR44] Monteleone P, Serritella C, Martiadis V, Maj M (2008). Decreased levels of serum brain-derived neurotrophic factor in both depressed and euthymic patients with unipolar depression and in euthymic patients with bipolar I and II disorders. Bipolar Disord.

[CR45] Moser D, Ekawardhani S, Kumsta R, Palmason H, Bock C, Athanassiadou Z, Lesch KP, Meyer J (2009). Functional analysis of a potassium-chloride co-transporter 3 (SLC12A6) promoter polymorphism leading to an additional DNA methylation site. Neuropsychoparmacol.

[CR46] Neves-Pereira M, Mundo E, Muglia P, King N, Macciardi, Kennedy JL (2002). The brain-derived neurotrophic factor gene confers susceptibility to bipolar disorder: evidence from a family-based association study. Am J Hum Genet.

[CR47] Numata S, Ye T, Hyde TM, Guitart-Navarro X, Tao R, Weinberger DR, Colantuoni C, Weinberger DR, Kleinman JE, Lipska BK (2012). DNA methylation signatures in development and aging of the human prefrontal cortex. Am J Hum Genet.

[CR48] Petronis A (2003). Epigenetics and bipolar disorder: new opportunities and challenges. Am J Med Genet C: Semin Med Genet.

[CR49] Petronis A, Gottesman II, Kan P, Kennedy JL, Basile VS, Paterson AD, Popendikyte V (2003). Monozygotic twins exhibit numerous epigenetic differences: clues to twin discordance?. Schizophr Bull.

[CR50] Proudfoot J, Doran J, Manicavasagar V, Parker G (2011). The precipitants of manic/hypomanic episodes in the context of bipolar disorder: a review. J Affect Disord.

[CR51] Pruunsild P, Kazantseva A, Aid T, Palm K, Timmusk T (2007). Dissecting the human BDNF locus: bidirectional transcription, complex splicing, and multiple promoters. Genomics.

[CR52] Psychiatric GWAS Consortium Bipolar Disorder Working Group (2011). Large-scale genome-wide association analysis of bipolar disorder identifies a new susceptibility locus near ODZ4. Nat Genet.

[CR53] Rao JS, Keleshian VL, Klein S, Rapoport SI (2012). Epigenetic modifications in frontal cortex from Alzheimer's disease and bipolar disorder patients. Transl Psychiatry.

[CR54] Rau TT, Rogler A, Frischauf M, Jung A, Konturek PC, Dimmler A (2012). Methylation-dependent activation of CDX1 through NF-kB: a link from inflammation to intestinal metaplasia in the human stomach. Am J Pathol.

[CR55] Schulze TG (2010). Genetic research into bipolar disorder: the need for a research framework that integrates sophisticated molecular biology and clinically informed phenotype characterization. Psychiatr Clin North Am.

[CR56] Scott LJ, Muglia P, Kong X, Guan W, Flickinger M, Upmanyu R, Tozzi F, Li JZ, Burmeister M, Absher D, Thompson RC, Francks C, Meng F, Antoniades A, Southwick AM, Schatzberg AF, Bunney WE, Barchas JD, Jones EG, Day R, Matthews K, McGuffin P, Strauss JS, Kennedy JL, Middleton L, Roses AD, Watson SJ, Vincent JB, Myers RM, Farmer AE (2009). Genome-wide association and meta-analysis of bipolar disorder in individuals of European ancestry. Proc Natl Acad Sci USA.

[CR57] Serretti A, Mandelli L (2008). The genetics of bipolar disorder: genome 'hot regions', genes, new potential candidates and future directions. Mol Psychiatry.

[CR58] Sklar P, Gabriel SB, McInnis MG, Bennett P, Lim YM, Tsan G, Schaffner S, Kirov G, Jones I, Owen M, Craddock N, DePaulo JR, Lander ES (2002). Family-based association study of 76 candidate genes in bipolar disorder: BDNF is a potential risk locus. Brain-derived neutrophic factor. Mol Psychiatry.

[CR59] Tadić A, Müller-Engling L, Schlicht KF, Kotsiari A, Dreimüller N, Kleimann A, Bleich S, Lieb K, Frieling H (2013). Methylation of the promoter of brain-derived neurotrophic factor exon IV and antidepressant response in major depression. Mol Psychiatry.

[CR60] Toledo-Rodriguez M, Lotfipour S, Leonard G, Perron M, Richer L, Veillette S, Pausova Z, Paus T (2010). Maternal smoking during pregnancy is associated with epigenetic modifications of the brain-derived neurotrophic factor-6 exon in adolescent offspring. Am J Med Genet B Neuropsychiatr Genet.

[CR61] Tramontina JF, Andreazza AC, Kauer-Sant’anna M, Stertz L, Goi J, Chiarani F, Kapczinski F (2009). Brain-derived neurotrophic factor serum levels before and after treatment for acute mania. Neurosci Lett.

[CR62] Vasilatos SN, Broadwater G, Barry WT, Baker JC, Lem S, Dietze EC, Bean GR, Bryson AD, Pilie PG, Goldenberg V, Skaar D, Paisie C, Torres-Hernandez A, Grant TL, Wilke LG, Ibarra-Drendall C, Ostrander JH, D'Amato NC, Zalles C, Jirtle R, Weaver VM, Seewaldt VL (2009). CpG island tumor suppressor promoter methylation in non-BRCA-associated early mammary carcinogenesis. Cancer Epidemiol Biomarkers Prev.

[CR63] Zhang AP, Yu J, Liu JX, Zhang HY, Du YY, Zhu JD, He G, Li XW, Gu NF, Feng GY, He L (2007). The DNA methylation profile within the 5′-regulatory region of DRD2 in discordant sib pairs with schizophrenia. Schizophr Res.

