# Supplementary material for: Quantitative leukocyte BDNF promoter methylation analysis in bipolar disorder
Source: Int J Bipolar Disord. 2013 Dec 30;1:28. doi: 10.1186/2194-7511-1-28 (PMC4215812; doi:10.1186/2194-7511-1-28)
Supplement: Supplementary file 1 — Additional file 1: Table S1: PCR, SAP and cleavage conditions, with primers. (DOC 34 KB) [file 40345_2013_26_MOESM1_ESM.doc]

**Additional flie 1: Table S1 PCR, SAP and cleavage conditions, with primers**

**1. Amplicons BDNF-D2a, BDNF-D3, BDNF-D5**

PCR Vol/rxn (μl)

Reagents

ddH20 1.00

10X HotStar Buffer 0.50

dNTPs (25 mM) 0.04

F primer (2 μM) 1.00

R primer (2 μM) 1.00

Qiagen HotStar Taq (5U/uL) 0.04

Bisulfite-Treated DNA 1.42

Total 5.00

**PCR-Meth**

95°C for 15 minutes

95°C for 20 seconds

56°C for 30 seconds 45 cycles

72°C for 1 minute

72°C for 3 minutes

10°C for ∞

Set up PCR in triplicates for each sample. After amplification, proceed to Shrimp Alkaline Phosphatase (SAP) treatment.

**2. SAP**

Reagent Vol/rxn (μl)

RNase-Free H2O 1.7

SAP (1.7U/uL) 0.3

Total 2.0

**SAP-Meth**

37°C for 20 minutes

85°C for 5 minutes

10°C for ∞

(SAP is part of the Sequenom MassCleave Kit)

Add 2.0 uL SAP master mix to each PCR well (2.0uL + 5.0 uL PCR). After SAP treatment, take 1 uL of SAP-treated PCR to run on a 1.5% agarose gel. Proceed to Cleavage RXN if PCR products are good.

**3. Transcription-Cleavage Run**

T -cleavage (Sequenom MassCleave Kit catalog # 10129)

Reagents Vol/rxn (μl)

RNase-Free ddH20 3.15

5X T7 Polymerase Buffer 0.89

T Cleavage Mix 0.24

DTT (100 mM) 0.22

T7 RNA & DNA Polymerase 0.44

RNase A (10 mg/mL) 0.06

Total Volume 5

Mix 5.0 uL cleavage mix with 2.0 uL SAP-treated PCR products. After incubation at 37°C for 3 hours , add 16 uL H2O and 6 mg clean Resin to each well. Rotate plate for 30 min, centrifuge for 5000rpm for 5min at room temperature. Spot analytes onto Sequenom chip.

**4. PCR Primers**

i) Sequence Name: BDNF-D2

Sequence:

AF411339.1 : from 1 to 115886 (BDNF gene: 32056…113952)

(from 34356-34855)

Sequence Length: 500

CpG island prediction results

(Criteria used: Island size > 100, GC Percent > 50.0, Obs/Exp > 0.6)

Island 1 338 bp (48 - 385)

Primer Start Size Tm GC% 'C's Sequence

Left primer 92 26 54.95 57.69 8 TTGGATAGAGTTATTAATTAGTTGGA

Right primer 378 25 54.75 52.00 8 CTCCCTAAAAAATAACAAAAAAATA

Product size: 287, Tm: 80.8, CpGs in product: 30

ii) Sequence Name: BDNF-D3

Sequence:

AF411339.1 : from 1 to 115886 (BDNF gene: 32056…113952)

(from 52660 - 53159)

Sequence Length: 500

CpG island prediction results

(Criteria used: Island size > 100, GC Percent > 50.0, Obs/Exp > 0.6)

2 CpG island(s) were found in your sequence

Size (Start - End)

Island 1 140 bp (59 - 198)

Island 2 219 bp (225 - 443)

Primer picking results for bisulfite sequencing (or restriction) PCR

Primer Start Size Tm GC% 'C's Sequence

Left primer 208 25 59.82 60.00 8 TTTTTTTTAGAGAATTTGGGTGTTG

Right primer 488 21 58.54 71.43 4 AAACCTATCCTCACCTCCTCC

Product size: 281, Tm: 73.5, CpGs in product: 23

iii) Sequence Name: BDNF-D5

Sequence:

AF411339.1 : from 1 to 115886 (BDNF gene: 32056…113952)

(from 53409 to 53959)

Sequence Length: 551

CpG island prediction results

(Criteria used: Island size > 100, GC Percent > 50.0, Obs/Exp > 0.6)

1 CpG island(s) were found in your sequence

Size (Start - End)

Island 1 211 bp (48 - 258)

Primer picking results for bisulfite sequencing (or restriction) PCR

Primer Start Size Tm GC% 'C's Sequence

Left primer 90 23 54.72 56.52 6 GGGGGTTTTAATGAGATATTTAT

Right primer 320 25 59.46 68.00 10 CTTTAAAATAAAAAATCCCCCAATC

Product size: 231, Tm: 77.2, CpGs in product: 19

CpG sites for each amplicon are displayed between Regions analyzed and the co-ordinates.
