# Supplementary material for: Quantitative leukocyte BDNF promoter methylation analysis in bipolar disorder
Source: Int J Bipolar Disord. 2013 Dec 30;1:28. doi: 10.1186/2194-7511-1-28 (PMC4215812; doi:10.1186/2194-7511-1-28)
Supplement: Supplementary file 2 — Additional file 2: Table S2: Methylation results by BDNF rs6265 genotype. (DOC 40 KB) [file 40345_2013_26_MOESM2_ESM.doc]

**Additional file 2: Table S2. Significance of methylation results by *BDNF* rs6265 genotype**

**Legend:** Z= Mann Whitney U test statistic

* Significant at alpha=0.05

****** Significant after FDR, alpha=0.004

Yellow= post hoc, significant at alpha<0.05

Orange= post hoc, significant at alpha<0.01

Genotypes: GG+AG+AA=all participants (n=100)

AG+AA= A(Met) allele carriers (n=33; 14 bipolar+19 controls)

GG= G (Val) allele homozygotes (n=66; 35 bipolar+31 controls)

na= genotype unavailable
